# Inspired, but not mimicking: a conversation between artificial intelligence and human intelligence

**DOI:** 10.1093/nsr/nwac068

**Published:** 2022-04-05

**Authors:** Weijie Zhao

**Affiliations:** NSR, news editor based Beijing, China

## Abstract

How intelligent is artificial intelligence (AI)? How intelligent will it become in the future? What is the relationship between AI and human intelligence (HI)? These questions have been a hot topic of discussion in recent years, but no consensus has yet been reached. To discuss these issues, we should first understand the concept of intelligence as well as the underlying mechanisms for both HI and AI. In this *NSR* Forum, experts from both disciplines gathered to discuss these issues; in particular, the similarities and differences between AI and HI, how these two disciplines could benefit from each other, and the emerging social and ethical challenges of AI.

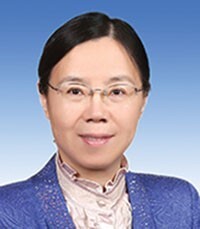

Xiaolan Fu

Professor at the Institute of Psychology, Chinese Academy of Sciences (CAS)

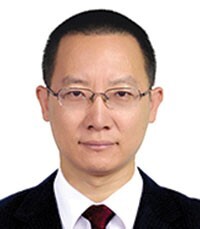

Yong Gu

Professor at the Institute of Neuroscience, Center for Excellence in Brain Science and Intelligence Technology, CAS

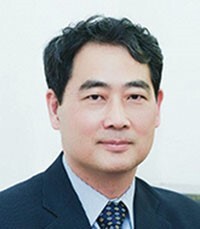

Sheng He

Professor at the Institute of Biophysics, CAS

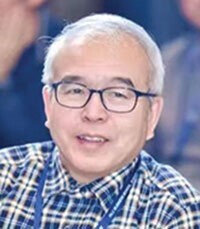

Zhuojun Liu

Professor at the Academy of Mathematics and Systems Science, CAS

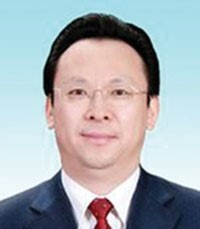

Tieniu Tan

Professor at the Institute of Automation, CAS

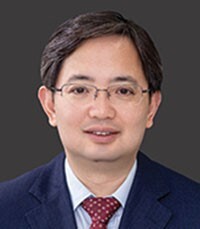

Zhi-Hua Zhou

Professor at National Key Laboratory for Novel Software Technology, Nanjing University

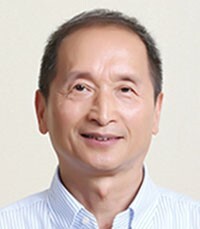

Huimin Lin (Chair)

Professor at the Institute of Software, CAS

**Lin:** Welcome to this panel discussion. I am a computer scientist, but not specialised in AI. AI, especially deep learning, has achieved great success in the past decade and many people in and out of this field have begun to think about the relationship between AI and HI. Here, we invite experts in neuroscience, cognitive science, psychology and AI to discuss various relevant issues.

## HUMAN INTELLIGENCE: A MULTIFACETED CONCEPT

**Lin:** First, what's human intelligence?

**He:** The concept and measurement of HI have been controversial for a long time. Loosely speaking, intelligence is the ability to acquire and use knowledge, including recognition, problem solving, etc. Historically, some researchers conceptualized that there could be a single index to measure intelligence. For example, Charles Spearman proposed his Theory of Intelligence in 1904, with the key idea that there is a single General factor (g factor) that underlies one's intelligence. Naturally, not everyone accepted this theory.

Other researchers tried to divide HI into different components. Robert Sternberg proposed the Triarchic Theory of Intelligence in the 1980s, suggesting that there are three types

Intelligence should contain two key components: knowledge, and the ability to obtain knowledge and use it to solve problems.

—Xiaolan Fu

of intelligence: analytical intelligence, creative intelligence and practical intelligence. These abilities are related but also distinct. For instance, some artists can be extremely creative, but they lack practical intelligence to deal with daily problems. This is likely related to the somewhat modular organization of the human brain, both structurally and functionally, to support different cognitive abilities.

So I think we can have a general definition of intelligence, but also more specialized definitions when it comes to specific problem-solving issues.

**Fu:** I think intelligence should contain two key components: knowledge, and the ability to obtain knowledge and use it to solve problems. These two components are the key abilities needed by all types of cognitive tasks.

**Gu:** I think intelligence is not just to have as much knowledge as possible, instead, it is to learn general rules from knowledge and apply them to new tasks. In neuroscience, there is a good example, which is the ‘cognitive map’ proposed by Edward Tolman in 1948. The concept was first proposed based on observations of rats’ behavior when they were wandering around a maze. During this spatial navigation task, rats first store a series of space and time events as egocentric coordinates to form ‘episodic memory’, which is further turned into more abstract ‘semantic memory’ in the form of a cognitive map. Based on the allocentric map, rats and other animals can use structured knowledge to navigate new environments, or plan new routes when certain paths are blocked in the maze.

Now, we know that a cognitive map is not only a map for spatial navigation, but also a map for abstract navigation, for example through a social, or value space. In a recent *Cell* article, scientists found that monkeys use the same brain areas, including the hippocampus, to navigate through a space, either physical or abstract. These brain areas are responsible for abstracting general laws and forming real knowledge that can be transferred to solve different problems. That is how humans and other animals possess the ability of meta learning, or learning to learn, which is really the key to intelligence, in particular the general intelligence that allows us to master multitasking.

**Lin:** Suppose we can create a machine to scan a person's brain and read the status and interconnections of all neurons. Can we then decode the knowledge he/she has learned, and the problems he/she could solve using that knowledge?

**Gu:** I think it's possible theoretically, but impossible practically. The human brain contains as many as 86 billion neurons, and each neuron can form as many as 1000 synapses with other neurons. So the possible structural combinations are nearly infinite. I do not think that we would be able to map all of that within 50 or 100 years.

**He:** It's impractical, but it may even be impossible theoretically. The human brain is extremely complex. Take a much simpler brain, such as the brain of a zebrafish; even if we could map all the neurons and synapses, based on our current understanding, we would still be unable to tell what knowledge or memory it contains.

## WHAT ABOUT THE INTELLIGENCE OF COMPUTERS?

**Lin:** Do computers have intelligence? What are the differences and similarities between computer intelligence and HI?

I would like to talk about it first. A computer is a machine created to perform computation. Since computation is an important aspect of human intelligence, computers do have intelligence. However, the intelligence of a computer is mechanical. Computers can only do what humans instruct them to do. They are fast and accurate, but do not have the abilities of creation and abstraction.

AI programs have been extremely successful. AlphaGo beat top human Go players SeDol Lee and Jie Ke, but it's only a program written by the DeepMind team members; it is a fruit human creativity. It has been proven to be impossible for computers to automatically generate programs from arbitrary requirements. Programs can only be designed and written by humans, because this needs creativity which computers do not possess. Computers are becoming more and more powerful because we have created more and more programs for them to run, but computers cannot extend their capabilities by themselves.

**Tan:** Since first being coined at the 1956 Dartmouth Conference, AI has been developing for 65 years. It has been an extremely hot topic in recent years, and has been listed as a national strategic priority by many countries. But we have to notice that its recent success is mostly due to breakthroughs in practical applications, especially the application of pattern recognition (such as face recognition), while on the theoretical side, there have not been major breakthroughs for a long time. Some people, especially those who are not real AI experts, are somehow over optimistic about AI.

Whether computers or machines possess intelligence or not depends on how we define intelligence. We can say that an AI system has intelligence in terms of the functional aspect of its behaviors, but essentially, the system does not really understand

[Computers] are fast and accurate, but do not have the abilities of creation and abstraction.

—Huimin Lin

what it is doing and why, so from this point of view, it seems that we cannot say it has intelligence.

**Zhou:** As an AI researcher coming from computer science discipline, I would like to say that machines can undoubtedly exhibit intelligent behaviors, such as reasoning and Go playing. However, it is hard to judge whether they can really have what the so-called ‘intelligence’. Indeed, we computer scientists do not care much about this, because our motivation, at least my own, is just to try to make some intelligent tools that can help people. I often take an analogy that we human beings saw bird flying in the sky and got inspiration to make aircrafts that can help us fly, but the flying mechanisms of aircrafts are very different from that of birds, and there is no need to demand aircrafts fly as same as birds.

**Liu:** In the 1992 textbook *Artificial Intelligence*, Patrick Winston of MIT gave a definition of AI: ‘Artificial Intelligence is the study of the computation that makes it possible to perceive, reason and act’. It means that AI is a technology that applies algorithms, models and other computational methods to realize some human intelligent behaviors, including receiving external information (looking and listening), reasoning (thinking) and exporting certain outputs (language and behavior). I think that is an appropriate understanding of AI, it is a tool inspired by the human brain and empowered by mathematical and computational methods that can realize multiple intelligent behaviors.

[AI] is a tool inspired by the human brain and empowered by mathematical and computational methods that can realize multiple intelligent behaviors.

—Zhuojun Liu

**Lin:** There is a famous statement in the first paragraph of *A Proposal for the Dartmouth Summer Research Project on Artificial Intelligence*: ‘The study is to proceed on the basis of the conjecture that every aspect of learning or any other feature of intelligence can in principle be so precisely described that a machine can be made to simulate it.’ But I think this conjecture is not true. For instance, a human's creative intelligent behavior cannot ‘be so precisely described that a machine can be made to simulate it’, and the behaviors that can be ‘precisely described’ must be mechanical which are exactly those can be done by computers.

**Fu:** I agree that computers have intelligence, but that intelligence is not as comprehensive as HI. With regard to knowledge, the knowledge that has been stored and mastered by computers is still quite limited, and does not contain knowledge of communication, sociality and emotion. With regard to the ability to obtain and use knowledge, there exists an essential difference between HI and AI: humans and animals need this ability to stay alive, but computers do not have that desire, they just follow the instructions.

## DO WE NEED STRONG AI WITH CONSCIOUSNESS AND EMOTION?

**Lin:** Is it necessary for AI to have consciousness and emotion?

**Zhou:** Computer scientists’ aim of developing AI is not to create human-like organisms, but to make intelligent tools that obey and help humans. From this aspect, there seems no need to try to create intelligent organisms that have self-consciousness, that may disobey or even take the place of human beings on this planet.

**Tan:** I agree. The aim of AI and other technologies is to enhance human capability and performance, not to completely replace humans. Many people are talking about strong AI or general-purpose AI. It's good to use one AI system for multiple tasks, but if general AI means an AI agent that can realize HI as a whole, I think it is neither possible nor necessary. We should not consider the development of such general AI systems as a major future research direction. In fact, it is sufficient and convenient to use specific-purpose AI algorithms to help us with different tasks.

**Fu:** But in some scenarios, if we hope the AI agent can integrate into human society and communicate and cooperate with human beings as a counterpart, it seems that they would need to have self-consciousness. They would need to recognize human emotion and give appropriate emotional responses. If they cannot do that, it would be impossible to achieve human–machine coexistence.

**Zhou:** There is a sub-direction of AI known as ‘affective computing’. Algorithms are being developed to make people feel that they are communicating with AI agents with emotions. However, these are just behaviors ‘exhibiting emotions’. It is hard to say that the agents really have the so-called ‘emotion’.

**Lin:** Technically, every activity a computer is able to perform is implemented by electronic circuits. A computer can execute a human-written program, to deceive people interacting with it that it has ‘emotion’, which does not mean the computer has emotion itself.

**He:** At this point, we do not yet have a good scientific definition for consciousness, so it would be difficult to discuss whether computers can have consciousness. I think we should first investigate the functions of human consciousness. Is consciousness a mere epiphenomenon of brain activity, or an important component of cognitive functions? In what way can consciousness benefit or enable cognitive functions?

If we can answer this question and list the functions, such as A, B and C, that require or depend on consciousness, but not function D, then we will have a better understanding of the role of consciousness in cognitive functions, so we can identify the benefits of consciousness (as seen in A, B and C) in AI.

**Gu:** That's right. Consciousness is not well defined. I think some better-defined parts of it are probably needed by AI. For example, the Theory of Mind says that in human society, people need to differentiate their own thoughts from the thoughts of ohters, and understand those thoughts. Autonomous driving probably needs this ability too. An autonomous vehicle needs to recognize the intentions of other vehicles to make appropriate decisions.

Another example is that robotic arms and other intelligent robots may need to have self-awareness of their own limbs, which can help them adapt to new tasks quickly and accurately. A recent study by a Columbia University group involved the construction of a robotic arm with 100 joints. It was asked to do random motion trajectories repeatedly. During this process, the robot learned to gradually build up an internal model of its arm status, and was able to perform the new task of grabbing balls and moving them to a desired cup with high accuracy without any feedback. Interestingly, modifying the arms could cause the robot to relearn and build up new internal models. This is quite like how human babies learn to develop self-awareness of their own arms, and when they get hurt, how to recover from it via plasticity.

Is consciousness a mere epiphenomenon of brain activity, or an important component of cognitive functions?

—Sheng He

## CONVERSATION BETWEEN THE TWO DISCIPLINES

**Lin:** The application of deep learning and pattern recognition has been a big success of AI. Machine-learning programs train classifiers with giant labeled data sets to perform recognition. Does the human brain use similar strategies to recognize objects?

**He:** Yes, there are some similarities between the structures of deep neural networks and the ventral occipitotemporal object-recognition pathway in the primate visual cortex. They are both hierarchical multileveled structures. The primary visual cortex processes line segments with different orientations; at the next stage, neurons represent configurations with medium complexity; then further upstream the information is classified into different categories such as cats and dogs, and in the case of human faces may eventually achieve individual recognition as somebody we know.

But there are also many differences. For example, there is extensive feedback modulation in human visual information processing. The current deep networks are primarily based upon feedforward signals, although there are efforts to incorporate feedback processing into the network. Human brains are very good at using contextual information or prior knowledge. If we see a fuzzy triangular shape in the kitchen, we may recognize it as a kitchen knife, but if we see the same shape in the bathroom, we may recognize it as a hairdryer. If we can make better use of feedback or contextual modulation in pattern recognition, it may help to avoid recognition errors caused by over-emphasizing local information such as textures.

Moreover, there are major parallel pathways in the human brain where different information is processed somewhat independently, which may help to prevent issues such as catastrophic forgetting when a network is trained sequentially on different tasks.

**Zhou:** The multilayered structure seems to be a similarity between deep neural networks and the human neural system, although multilayered structure has been used in neural networks for a long time. But until 2005 people did not know how to train a neural network with more than five layers, because gradient vanishing with more layers. The deep learning breakthrough started from a computational trick: to do one layer each time and then pining them together for global refining.

**Gu:** Besides recognition, AI systems and the human brain have similarities in spatial navigation and autonomous driving. My lab studies multisensory integration, in particular how the brain integrates visual and vestibular information for effective self-motion perception during locomotion or spatial navigation. Similar ideas have been used in autonomous driving systems, which integrate information from multiple sources including GPS, inertial motion unit, video cameras and radars to help drive under complex and dynamic scenarios.

Another example is the grid cells that were discovered in 2005 by the 2014 Nobel Prize winners May-Britt Morser and Edvard Morser in the entorhinal cortex of rodents. The discovery of grid cells led to a flurry of computational work aiming to understand their function in navigation. For example, in 2018, DeepMind published a paper in *Nature* showing that for a recurrent neural network with reinforcement learning, ‘grid-like cells’ appeared in the hidden layer after being trained with rats’ behavioral data. Interestingly, the appearance of these ‘AI grid cells’ significantly improved the overall performance of the network by exhibiting animal and human-like intelligence during navigation in new environments, or environments with changed contexts like blocked paths. Thus, the brain hippocampal-entorhinal system provides a very useful guide for AI in spatial navigation tasks.

**Lin:** The artificial neural network was proposed in the early 1940s, and tremendous progresses have been made in neural science since then. Now, to what extent is an AI system similar to a human nervous system?

**He:** I think they are more different than similar.

Compared with biological nervous systems, artificial neural networks are still over-simplified models.

—Yong Gu

**Gu:** Yes. Compared with biological nervous systems, artificial neural networks are still over-simplified models. In a human nervous system, other than typical bottom-up projections, neurons within a layer form many lateral connections. There are also many feedback top-down projections. In the brain, there are also many different types of neurons—excitatory neurons, inhibitory neurons and their subgroups with different shapes and projected targets, indicating heterogeneous functions. There are also many different types of neurotransmitters—dopamine, serotonin, norepinephrine etc.—that affect the brain's state and information processing. Many of these traits have not been implemented in AI systems.

It is said that only 10% of human brain potential has been exploited, and I think only 10% of the human brain, or even less, has been simulated by AI. So I am very optimistic about AI, in the sense that it still has a huge space to develop.

**Lin:** Have AI researchers tried to mimic and make use of these complex structures and functions of human brain?

**Zhou:** AI researchers are anxious to get inspiration from neuroscience, and there were successful stories. For example, the MP-model proposed in 1943, inspired by neuron mechanisms, is still the basis of almost all current deep neural networks. However, in most cases, direct mimicking is quite difficult to get success. For example, there were efforts trying to mimic the pulse-spiking mechanism of neurons, where not only the potential but also peak time are considered. Though it offers advantages from biological plausible aspect, after half a century's exploration, the algorithmic success is very limited and more explorations are needed.

Generally, it is often very difficult to transform neuroscience findings directly into effective computer algorithms, and the return is often marginal. It seems that the most important thing AI researchers can get from neuroscience would be directional inspirations, motivating us about what things can be tried to tackle. While as for how to tackle them, we usually need resort to mathematics and engineering approaches, rather than trying to direct mimicking of the human brain.

It seems that the most important thing AI researchers can get from neuroscience would be directional inspirations, motivating us about what things can be tried to tackle.

—Zhi-Hua Zhou

**Lin:** We talked about brain science as being an inspiration for AI. What about the other direction? Can AI help with brain science?

**Gu:** AI can help brain science research in at least two aspects. Firstly, AI offers a great tool. For example, it can help with image recognition work in the construction of mesoscopic neural connection maps with high efficiency and high speed. AI also helps doctors to analyze medical scanned images.

Secondly, I think AI can in turn help us understand the human brain. I believe that HI is only one of the many possible forms of intelligence, which is generated in specific environments after a long period of evolution. By analyzing AI systems and comparing them with the human brain, we have a chance to see the possible mechanisms involved in forming intelligence that are different from the human brain. We have seen some hints in AlphaGo Zero, which is the second version of AlphaGo. With reinforcement learning, AlphaGo Zero did not need any training data set from humans. Instead, it learned by playing Go with itself. After only a few days, it not only defeated top human Go players but also created some moves that had never been thought of by humans. Because of this, AI is now used to train human professionals, improving their chess ability much faster than a human coach could. I think we should appreciate this difference in strategy between AI and HI. If we can further analyze these differences, it will also help us to better understand the functions of the human brain and why the human brain works the way it does.

**Lin:** I have a few more comments about AlphaGo Zero. A Go board has 361 grid points, so there are as many as 3^361^ possible configurations, which cannot be exhausted by the world's fastest computer in reasonable time. But to determine the winner, it needs only to count the 361 points, which can be accomplished by a personal computer in no time. So it's easy for AlphaGo Zero to play with itself, record the strategies of both sides, and at the end of a game, keep the winner's strategies and discard the loser's. But this approach is applicable for very few problems.

**He:** Besides, we can also use AI as a ‘sand table’ for modeling the nervous system. It is more flexible and costs less than using animal models. By testing its process and observing different outputs under different inputs, we could gain some insight into neuroscience questions.

## THE SOCIAL AND ETHICAL CHALLENGES OF AI

**Lin:** What are the possible social and ethical problems that may be caused by AI?

**Tan:** This has been a long-discussed issue. I think one of the most urgent problems is Deepfake, or automatic content generation. AI is already able to generate text, voice, image, video and other contents with an incredibly high level of realism. If we cannot strictly control its usage, it will present real risks for public security and national security. There are also other social problems that may be caused by the use of AI, such as privacy issues, employment issues and issues of equality—how can different nations, organizations and individuals get equal access to AI technologies, and avoid an intelligence divide?

Researchers are also trying to keep AI controllable and avoid these problems. Some are developing algorithms to identify deepfake content. There are also technologies that can encode

Whether AI is a devil or an angel depends on whether the person using it is a devil or an angel.

—Tieniu Tan

and transform collected private data, such as biometric data, before its usage, so as to avoid privacy disclosure and personal information leakage.

We should take both technical and managerial measures to deal with these challenges. The good news is that China and many other countries have already started to address these issues and are beginning to set up supervisory measures. Challenges are inevitable, and the most important thing is to formulate necessary laws and regulations in time.

Actually, like AI, the development of other technologies would also bring similar challenges. Whether AI is a devil or an angel depends on whether the person using it is a devil or an angel. So our researchers should be responsible—that is essential to guaranteeing that the tools we make are controllable and beneficial.

**Liu:** It is important to keep AI controllable. We cannot authorize algorithms to make fundamental decisions, such as to launch a guided missile or not. I have talked with some companies and helped them to create standards with regard to AI. I think it's important to have companies involved in the process of policy making, so that we can better supervise them and promote the healthy development of the AI industry at the same time.

**Fu:** Many end users worry a lot about privacy risks, some are even afraid of the possibility that stronger machines will harm people.

**Zhou:** As I have mentioned, most AI researchers just want to make intelligent tools that can help human beings, rather than man-made intelligent organisms coming with big risks or even may take place of human beings on the planet. This, however, is not that clear to the public, and thus, it is possible to make people feel threatened. So, we should make more communication efforts to help the public get a better understanding of AI research.

**Gu:** AI chatbots are developing fast, but an emerging problem is that they may learn ‘bad words’ or improper world views from a too-large data set that lacks supervision. As a result, they may talk in a violent or racially discriminative way. It is a big challenge to keep these chatbots controllable.

**Zhou:** This is a challenge for current technology. The chatbots generally learn from extremely huge corpuses, and it is hard to have enough human resources to screen and clear those huge corpuses in advance.

**Tan:** It should be AI researchers’ responsibility to cope with this issue. We should develop more efficient methods for selecting and building a corpus.

**He:** There are issues that may not pose an immediate danger, but should also be considered. For example, should we apply AI to every task that it is capable of? For some industries, the application of AI may cause mass unemployment. Should we slow down AI application in these fields? Moreover, AI gets seemingly better and better at creating literature and art. Algorithms can paint pictures, write poems and compose music. Should we encourage AI in these activities? Or should we leave those creative works for ourselves, so that we can enjoy the fun of creation? These are questions that we should consider.

**Lin:** Thanks for the discussion today on HI and AI, their relationship, and the social and ethical challenges regarding AI. Such interdisciplinary communication is thought-provoking and can expand our vision.

